# Pan Arab Osteoporosis Society Guidelines for Osteoporosis Management

**DOI:** 10.31138/mjr.28.1.27

**Published:** 2017-03-28

**Authors:** Nizar Abdulateef Jassim, Gemma Adib, Younis Ali Abdul Rahman, Faiq Isho Gorial, Abdullah Maghraoui, Abdul Rahim Al Suhaili, Ahmad Murtaji, Ali Otom, Basel Masri, Elias Saba, Farid Badran, Ghassan Maalouf, Jamal Saleh, Khaled El Muntaser, Leith Zakraoui, Mustafa Al Izzi, Nadia Al Ali, Riad Sulaimani, Said Abdul Majeed, Samar Al Emadi

**Affiliations:** 1 Iraqi Osteoporosis Society,; 2 Syrian National Osteoporosis Society,; 3 Moroccan Society for Rheumatology,; 4 Emirates Osteoporosis Society,; 5 Egyptian Osteoporosis Prevention Society,; 6 Jordanian Physicians Osteoporosis Society,; 7 Jordanian Osteoporosis Prevention Society,; 8 Palestinian Osteoporosis Prevention Society,; 9 Lebanese Osteoporosis Prevention Society,; 10 Bahrain Osteoporosis Society,; 11 Libyan Osteoporosis Prevention Society,; 12 Tunisian Osteoporosis Prevention Society,; 13 Kuwaiti Osteoporosis Society,; 14 Saudi Osteoporosis Society,; 15 Hamad Medical Corporation

**Keywords:** Pan Arab, Guidelines, Osteoporosis

## Abstract

Osteoporosis is the most common bone disease in humans. With its related fragility fracture, it represents a major public health problem in our region, with a significant medical and socio-economic burden.

The high prevalence rate of vitamin D deficiency, the increase in life expectancy, the low socioeconomic level and the significant restriction to access to health care in some countries represent the major causes for the increasing prevalence of osteoporosis and incidence of fragility fractures in the Arabic countries.

Bone mineral density (BMD) assessment is the gold standard to diagnose osteoporosis. However, a clinical diagnosis of osteoporosis may be made in the presence of a fragility fracture, without BMD measurement. Dual energy x-ray absorptiometry (DXA) is the preferred method for screening bone mineral density. For screening site of measurement, DXA of hip and spine is suggested. BMD assessment is recommended in all women 65 years of age and older and men 70 and older regardless of risk factors. Younger subjects with clinical risk factors and persons with clinical evidence of osteoporosis or diseases leading to osteoporosis should also be screened.

These guidelines are aimed to provide to health care professionals in the region of an updated process for the diagnosis and treatment of osteoporosis. It includes risk factors for osteoporosis and the indications for screening, diagnosis of osteoporosis, treatment of osteoporosis in postmenopausal and premenopausal women, and men; in addition to prevention and treatment of glucocorticoid-induced osteoporosis.

## OSTEOPOROSIS

Osteoporosis is the most common bone disease in humans, representing a major health problem.^1^ It is characterized by low bone mass, deterioration of bone tissue and disruption of bone architecture, compromised bone strength, and an increase in the risk of fracture. The risk of fractures is highest in those with the lowest bone mineral density (BMD); however, the majority of fractures occur in patients with low bone mass (osteopenia) rather than osteoporosis, because of the large number of individuals with bone mass in this range. There is an estimated nine million of osteoporotic fractures worldwide in 2000.^2^ Some estimates predict a continued increase in the number of hip fractures over the next 40 years.^3^

## OSTEOPOROSIS SCREENING

### Risk factor screening

Assessing risk factors for osteoporosis and fracture in all postmenopausal women and men age 50 and older is recommended to identify those at high risk for fractures.^4^ The principal BMD-independent risk factors to consider include advanced age, previous fragility fracture, glucocorticoids, risk of falls, smoking, alcohol, low body weight, family history of fracture, rheumatoid arthritis, and secondary osteoporosis (e.g. hypogonadism or premature menopause, malabsorption, chronic liver disease, and inflammatory bowel disease).^5^

### Bone mineral density screening

- BMD assessment is recommended in:^6–8^
  1. All women 65 years of age and older and men 70 and older regardless of risk factors;
  2. Postmenopausal women younger than 65 and men 50 to 70 years if one of the above risk factors is present;
  3. Routine BMD measurements in premenopausal women and men younger than 50 years is not recommended. BMD testing is recommended in the following conditions:
    - History of fragility fracture.
    - Diseases, conditions, or medications known to be associated with low bone mass or bone loss.
- For screening BMD, Dual energy x-ray absorptiometry (DXA) is preferred over peripheral measurements.
- Regarding skeletal site to measure, DXA of hip and spine is suggested. However, measurement of the hip alone could be sufficient in older individuals.^9,10^
- The 33 percent forearm (1/3 radius) site is recommended in the following cases:^11^
  1. If hip and/or spine cannot be measured or interpreted;
  2. Hyperparathyroidism;
  3. Severe obesity (over the weight limit of DXA table).

### Repeating BMD measurements^12,13^

- Repeating BMD measurements may be most valuable for individual patients on therapy or with underlying clinical conditions that might lead to accelerated bone loss.
- In women and men with advanced osteopenia (T-score −2.00 to −2.49) or who have risk factors for ongoing bone loss (e.g., glucocorticoid use, hyperparathyroidism), follow-up measurements (approximately every one to two years) is suggested, as long as the risk factor persists.
- In women 65 years of age and older at baseline screening, with moderate osteopenia (T-score −1.50 to −1.99), and with no risk factors for accelerated bone loss, a follow-up DXA in three to five years is suggested.
- In women 65 years of age and older with normal BMD or mild osteopenia (T-score greater than −1.5) at baseline measurement and no risk factors for accelerated bone loss, a follow-up DXA in 10 to 15 years is suggested.

## DIAGNOSIS OF OSTEOPOROSIS

A clinical diagnosis of osteoporosis may be made in the presence of a fragility fracture, regardless of BMD. A fragility fracture has been defined as one that occurs after a fall from the standing position or one that is associated with low BMD and increases in incidence with age.^14^

The World Health Organization (WHO) has established a classification of BMD according to T-score that has been adapted for clinical use by the International Society for Clinical Densitometry (ISCD) (**Table 1**).^15,16^

**Table 1.** WHO Classification of BMD according to T-score.

Normal	≥−1.0
Low bone mass (osteopenia)	Between −1.0 and −2.5
Osteoporosis	≤−2.5
Severe (established) osteoporosis	≤−2.5 and fragility fracture

The diagnosis of osteoporosis in clinical practice can be made by DXA using the lowest T-score of the lumbar spine (L1–L4), total proximal femur, or femoral neck.^9,10^ In the hip, Ward’s area, trochanter, and other regions of interest (ROIs) should not be used for diagnosis.

In postmenopausal women, The WHO classification of BMD may be applied to perimenopausal and postmenopausal women of all ethnicities.^15^ The WHO selected a T-score of −2.5 or less to define osteoporosis.

However, in premenopausal women, Z-scores, not T-scores, should be used in premenopausal women. A clinical diagnosis of osteoporosis may be made in the presence of a fragility fracture, or when there is low BMD (Z-score ≤−2.0) and risk factors for fracture, such as long-term glucocorticoid therapy or hyperparathyroidism.^17^

In men, the WHO classification may be used in men age 50 and older.^18^ In younger men, Z-scores, not T-scores, should be used, and a clinical diagnosis of osteoporosis may be made in the presence of a fragility fracture, or when there is low BMD (Z-score ≤−2.0) and risk factors for fracture.^10^

In children, the WHO classification should not be used in children and adolescents. The diagnosis of osteoporosis in children and adolescents requires the presence of both a clinically significant fracture history and low bone mineral content (BMC) or BMD. A clinically significant fracture history is one or more of the following: long bone fracture of the lower extremities, vertebral compression fracture, or two or more long bone fractures of the upper extremities. Low bone mineral content or bone mineral density is defined as a BMC or a real BMD Z-score that is less than or equal to −2.0, adjusted for age, gender, and body size, as appropriate.^19^

## FRACTURE RISK ASSESSMENT

Using the Fracture Risk Assessment Tool (FRAX) is recommended to guide treatment in patient with osteopenia. FRAX estimates the 10-year probability of hip fracture and major osteoporotic fracture for an untreated patient (40 to 90 years of age) using femoral neck BMD (g/cm^2^) and easily obtainable clinical risk factors for fracture (file://www.shef.ac.uk/FRAX/).^20,21^

## INITIAL EVALUATION

The evaluation typically begins with history, physical examination, and basic biochemical testing. A detailed history and a focused physical examination are recommended to identify risk factors for low BMD, falls and fractures, as well as undiagnosed vertebral fractures. Lifestyle factors that contribute to bone loss, including smoking, excessive alcohol, physical inactivity, and poor nutrition, should be addressed. Height and weight should be measured.

Initial laboratory studies should include complete blood count, biochemistry profile (especially calcium, phosphorous, albumin, total protein, creatinine, liver enzymes, including alkaline phosphatase, and electrolytes), 25-hydroxyvitamin D, and assessment of urinary calcium excretion.^22,23^

The presence of abnormalities on initial laboratory testing, suspicious findings on history and physical examination suggesting a secondary cause of osteoporosis, or Z-scores ≤ −2.0 indicate additional evaluation for these secondary causes.^24^

## MANAGEMENT OF OSTEOPOROSIS IN POSTMENOPAUSAL WOMEN

### Non-pharmacologic therapy

- Adequate calcium and vitamin D is recommended for all postmenopausal women with osteoporosis. In general, 1200 mg of elemental calcium daily, total diet plus supplement, and 800 international units of vitamin D daily is suggested. Some patients require additional vitamin D supplementation.^25^
- Important additional lifestyle measures include exercise,^26,27^ counseling on fall prevention,^28^ smoking cessation,^29,30^ and avoidance of heavy alcohol use^31^ for all postmenopausal women with osteoporosis.

### Pharmacologic therapy

- In addition to non-pharmacologic therapy, pharmacologic therapy is recommended for postmenopausal women with established osteoporosis (T-score ≤−2.5) or fragility fracture (hip or vertebral).^32^
- For the treatment of postmenopausal women with T-scores between −1.0 and −2.5, pharmacologic therapy is suggested if the 10-year probability of hip fracture or combined major osteoporotic fracture of ≥3.0 or ≥20 percent, respectively.^32^ If FRAX score is not available, treatment is suggested for women more than 75 years old, or for those with 2 or more clinical risk factors.^33^
- For the treatment of osteoporosis in postmenopausal women, bisphosphonates are suggested as first-line therapy. Oral bisphosphonates are preferred as initial therapy because of their efficacy, cost effectiveness, and long-term safety data.^34–40^
- For most postmenopausal women with osteoporosis, alendronate or risedronate are preferred over oral ibandronate. Although oral ibandronate may be more convenient for patients, a reduction in the risk of hip fracture has not been established in randomized trials.^41–44^
- An intravenous bisphosphonate formulation is suggested for patients who cannot tolerate oral bisphosphonates or who have difficulty with dosing requirements, including an inability to sit upright for 30 to 60 minutes. Zoledronic acid is the only intravenous bisphosphonate that has demonstrated efficacy for fracture prevention^45^ and is, therefore, suggested as intravenous bisphosphonate of choice.
- Denosumab is suggested as an alternative option for the treatment of postmenopausal women with osteoporosis at high risk for fracture (history of osteoporotic fracture, multiple risk factors for fracture) who have failed or are intolerant to other available osteoporosis therapies) and for those who have impaired renal function.^46–48^
- Raloxifene is suggested for postmenopausal women with osteoporosis (low bone mineral density [T score <−2.5] and no fragility fractures) who cannot tolerate or are not candidates for any bisphosphonates or for postmenopausal women with osteoporosis who are also at high risk for invasive breast cancer.^49,50^
- Parathyroid hormone (teriparatide) therapy is suggested in the following situations:^51,52^
  1. Postmenopausal women with severe osteoporosis (low bone mineral density [T score <−2.5] and at least one fragility fracture) who are unable to tolerate any of the available bisphosphonates.
  2. Postmenopausal women with severe osteoporosis (low bone mineral density [T score <−2.5] and at least one fragility fracture) who continue to fracture after one year of bisphosphonate therapy.
  3. Postmenopausal women with osteoporosis who are unable to tolerate bisphosphonates, or who have relative contraindications to bisphosphonates (achalasia, scleroderma esophagus, esophageal strictures), in addition to relative contraindications to raloxifene (thrombosis, hot flashes), or for whom other osteoporosis therapies fail.

## MONITORING

For patients starting on therapy, we suggest a follow-up DXA of the hip and spine after one to two years, and if BMD is stable or improved, less frequent monitoring thereafter.^53^

## MANAGEMENT OF PREMENOPAUSAL OSTEOPOROSIS

- Adequate calcium, vitamin D, and exercise are recommended in all premenopausal women with low bone mass. Elemental calcium (from diet and supplements) of 1000 mg and 600 IU vitamin D daily is recommended.
- In women with a secondary cause of low BMD, treatment should be targeted to that cause.
- For premenopausal women of childbearing age potential taking or initiating glucocorticoids who have amenorrhea, oral contraceptive or hormone replacement therapy (if not contraindicated) is suggested. Bisphosphonates are an option for women with fragility fractures or accelerated bone loss (≥4 percent/year) while receiving glucocorticoids (7.5 mg prednisone equivalent for ≥3 months) who do not need estrogen replacement therapy (normal menstrual function).^54^ Teriparatide is an alternative option for such women, as long as epiphyses are fully fused.^55,56^
- For women with chemotherapy-induced early menopause with low BMD (T-score <−2.5) and/or fragility fracture, bisphosphonates are suggested.^57–59^
- Conservative management without specific medical therapy is suggested for premenopausal women with isolated low bone mass (no fragility fractures and no known secondary cause). These women should have a repeat bone density measurement in one year to ensure stable BMD.

## TREATMENT OF OSTEOPOROSIS IN MEN

The treatment of osteoporosis in men includes lifestyle measures, calcium and vitamin D supplementation, and hormonal or pharmacologic therapy.

- In men with osteoporosis, calcium and vitamin D supplementation is recommended. 1000 to 1200 mg of calcium (total diet plus supplement) and 600 to 800 IU of vitamin D daily are generally suggested. The dose of calcium and vitamin D may vary in individuals with coexisting medical conditions.
- For men with osteoporosis and symptomatic hypogonadism and who do not have any contraindications to testosterone therapy, testosterone replacement therapy is indicated.
- For hypogonadal men who are at high risk for fracture, the addition of pharmacologic therapy to testosterone therapy is suggested.^60^ High risk groups might include hypogonadal men whose BMD T-score is <−2.5 even after receiving adequate testosterone replacement therapy for two years; men on high-dose glucocorticoids; men with frequent falls; men who have had a recent fragility fracture, particularly if they have a BMD T-score below −2.5 at any skeletal site; or men with T-scores below −3.5 or even below −3 if they have other risk factors for fracture.
- For the treatment of men with osteoporosis (T-score below −2.5 or fragility fracture) who do not have symptomatic hypogonadism or in hypogonadal men in whom testosterone therapy is contraindicated, pharmacologic therapy is recommended.
- For the treatment of high risk men with T-scores between −1.0 and −2.5, pharmacologic therapy is suggested. A reasonable cut point that may be cost-effective in some settings is a 10-year probability of hip fracture or combined major osteoporotic fracture of ≥3.0 or ≥20 percent, respectively.
- Choice of pharmacologic therapy: men seem to respond to available therapies in the same way that women respond, so that, the approach to treating men and women with osteoporosis is quite similar.

## PREVENTION AND TREATMENT OF GLUCOCORTICOID-INDUCED OSTEOPOROSIS

### General principles

- Glucocorticoid dose and the duration of therapy should be as low and as short as possible, because even replacement doses can cause bone loss.^61^ Patients should be encouraged to do weight-bearing exercises for protection against both bone loss and muscle atrophy. Patients should avoid smoking and excess alcohol and take measures to prevent falls.
- For all patients receiving any dose of chronic glucocorticoid therapy or initiating glucocorticoids with an anticipated duration of ≥3 months, calcium and vitamin D supplementation is recommended.^54^ Most individuals require 1200 mg of elemental calcium daily, total diet plus supplement, and 800 IU of vitamin D daily.^54^

## CANDIDATES FOR PHARMACOLOGIC THERAPY

We adopt the Royal College of Physicians guidelines which recommends beginning antiresorptive therapy at the time of starting glucocorticoids (expected duration 3 months or more) in patients ≥65 years of age, or history of prior fragility fracture. Bone density testing should be considered in other individuals who will be receiving glucocorticoids for ≥3 months, and therapy considered for those with a T-score of ≤−1.5 or with a reduction of BMD >4 percent after one year.^62^

## MONITORING

BMD is measured at the initiation of glucocorticoid therapy and after one year. If BMD is stable or improved, we measure BMD less frequently (every two to three years) thereafter.

## CHOICE OF THERAPY

For pharmacologic therapy for men and postmenopausal women, bisphosphonates is considered as first-line therapy. Alendronate or risedronate are preferred because of clinical trial data demonstrating efficacy in men and women with glucocorticoid-induced osteoporosis. For patients who cannot tolerate oral bisphosphonates or who have difficulty with the dosing requirements, intravenous zoledronic acid is a good alternative. Parathyroid hormone (teriparatide) is an option for patients who are unable to tolerate any of the available bisphosphonates or who continue to fracture after one year of bisphosphonate therapy.

For premenopausal women with fractures or accelerated bone loss who do not need estrogen replacement therapy (normal ovarian function), bisphosphonates are generally the drugs of choice. Teriparatide is an alternative option for such women, as long as epiphyses are fully fused.

## References

[1] Office of the Surgeon General (US) (2004) Bone health and osteoporosis: a report of the Surgeon General. Office of the Surgeon General (US), Rockville (MD). 20945569

[2] Johnell O Kanis J A An estimate of the worldwide prevalence and disability associated with osteoporotic fractures. Osteoporos Int 2006;17:1726. 16983459 10.1007/s00198-006-0172-4

[3] Gullberg B Johnell O Kanis J A World-wide projections for hip fracture. Osteoporos Int 1997;7:407. 9425497 10.1007/pl00004148

[4] Clinician’s guide to prevention and treatment of osteoporosis. National Osteoporosis Foundation http://nof.org/hcp/resources/913. Accessed Nov. 20, 2014. 10.1007/s00198-014-2794-2 PMC4176573 25182228

[5] Kanis J A Borgstrom F De Laet C Assessment of fracture risk. Osteoporos Int 2005;16:581. 15616758 10.1007/s00198-004-1780-5

[6] NOF’s New Clinician’s Guide to Prevention and Treatment of Osteoporosis http://www.nof.org/sites/default/files/pdfs/NOF_ClinicianGuide2009_v7.pdf (Accessed on January 10, 2011).

[7] The Internationals Society for Clinical Densitometry Official Positions www.iscd.org/Visitors/positions/OfficialPositionsText.cfm (Accessed on January 10, 2011).

[8] Lewiecki EM Gordon CM Baim S International Society for Clinical Densitometry 2007 Adult and Pediatric Official Positions. Bone 2008;43:1115. 18793764 10.1016/j.bone.2008.08.106

[9] National Osteoporosis Foundation Clinician’s Guide to Prevention and Treatment of Osteoporosis. Washington, DC: National Osteoporosis Foundation, 2010 www.nof.org/sites/default/files/pdfs/NOF_ClinicianGuide2009_v7.pdf (Accessed on June 14, 2012).

[10] The International Society for Clinical Densitometry Official positions www.iscd.org/Visitors/positions/OfficialPositionsText.cfm (Accessed on June 01, 2008).

[11] International Society for Clinical Densitometry 2013 Official Positions—Adult. http://www.iscd.org/official-positions/2013-iscd-official-positions-adult/. Accessed Feb 2014.

[12] Osteoporosis: diagnosis, treatment and fracture prevention [Internet]. Vancouver (BC): British Columbia Medical Services Commission; 2011 5 1 [revised 2012 Oct 1; cited 2015 Mar 3].

[13] Gourlay M L Fine J P Preisser J S May R C Li C Liu L Y Study of Osteoporotic Fracture Research Group Bone-density testing interval and transition to osteoporosis in older women. N Eng J Med 2012;366:225–33. 10.1056/NEJMoa1107142 PMC3285114 22256806

[14] Kanis J A Oden A Johnell O The burden of osteoporotic fractures: a method for setting intervention thresholds. Osteoporos Int 2001;12:417. 11444092 10.1007/s001980170112

[15] Baim S Binkley N Bilezikian J P Official Positions of the International Society for Clinical Densitometry and executive summary of the 2007 ISCD Position Development Conference. J Clin Densitom 2008;11:75. 18442754 10.1016/j.jocd.2007.12.007

[16] Kanis J A on behalf of the World Health Organization Scientific Group (2007). Assessment of osteoporosis at the primary healthcare level. Technical Report. World Health Organization Collaborating Centre for Metabolic Bone Diseases, University of Sheffield, UK 2007: Printed by the University of Sheffield. www.shef.ac.uk/FRAX/pdfs/WHO_Technical_Report.pdf (Accessed on November 02, 2010).

[17] Writing Group for the ISCD Position Development Conference Diagnosis of osteoporosis in men, premenopausal women, and children. J Clin Densitom 2004;7:17. 14742884 10.1385/jcd:7:1:17

[18] Kanis J A McCloskey E V Johansson H A reference standard for the description of osteoporosis. Bone 2008;42:467. 18180210 10.1016/j.bone.2007.11.001

[19] Baim S Leonard M B Bianchi M L Official Positions of the International Society for Clinical Densitometry and executive summary of the 2007 ISCD Pediatric Position Development Conference. J Clin Densitom 2008;11:6. 18442749 10.1016/j.jocd.2007.12.002

[20] Kanis J A Johnell O Oden A FRAX and the assessment of fracture probability in men and women from the UK. Osteoporos Int 2008;19:385. 18292978 10.1007/s00198-007-0543-5 PMC2267485

[21] WHO Fracture Risk Assessment Tool (FRAX) www.shef.ac.uk/FRAX (Accessed on June 05, 2012).

[22] Hodgson S F Watts N B Bilezikian J P American Association of Clinical Endocrinologists medical guidelines for clinical practice for the prevention and treatment of postmenopausal osteoporosis: 2001 edition, with selected updates for 2003. Endocr Pract 2003;9:544. 14715483 10.4158/EP.9.6.544

[23] Tannenbaum C Clark J Schwartzman K Yield of laboratory testing to identify secondary contributors to osteoporosis in otherwise healthy women. J Clin Endocrinol Metab 2002;87:4431. 12364413 10.1210/jc.2002-020275

[24] Lewiecki E M Watts N B McClung M R Official positions of the international society for clinical densitometry. J Clin Endocrinol Metab 2004;89:3651. 15292281 10.1210/jc.2004-0124

[25] Institute of Medicine Report at a Glance, Report Brief: Dietary Reference Intakes for Calcium and Vitamin D, released 11/30/2010.

[26] Granacher U Gollhofer A Hortobágyi T Kressig R W Muehlbauer T The importance of trunk muscle strength for balance, functional performance and fall prevention in seniors: a systematic review. Sports Med 2013;43:627–641. 23568373 10.1007/s40279-013-0041-1

[27] Sherrington C Whitney J C Lord S R Herbert R D Cumming R G Close J C Effective exercise for the prevention of falls: a systematic review and meta-analysis. J Am Geriatr Soc 2008;56:2234–43. 19093923 10.1111/j.1532-5415.2008.02014.x

[28] Choi M Hector M Effectiveness of intervention programs in preventing falls: a systematic review of recent 10 years and meta-analysis. J Am Med Dir Assoc 2012;13:188.13–21. 10.1016/j.jamda.2011.04.022 21680249

[29] Ayo-Yusuf O A Olutola B G Epidemiological association between osteoporosis and combined smoking and use of snuff among South African women. Niger J Clin Pract 2014;17:174–7. 24553027 10.4103/1119-3077.127542

[30] Waugh E J Lam M A Hawker G A McGowan J Papaioannou A Cheung A M Risk factors for low bone mass in healthy 40–60 year old women: A systematic review of the literature. Osteoporos Int 2009;20:1–21. 18523710 10.1007/s00198-008-0643-x PMC5110317

[31] Mikosch P Alcohol and bone. Wien Med Wochenschr 2014;164:15–24. 24477631 10.1007/s10354-013-0258-5

[32] National Osteoporosis Foundation Clinician’s Guide to Prevention and Treatment of Osteoporosis. www.nof.org/sites/default/files/pdfs/NOF_Clinicians_Guide2008

[33] Hough S Evans B H A Brown S L NOFSA Guideline for the Diagnosis and Management of Osteoporosis. JEMDSA 2010;15(3).

[34] Tucci J R Tonino R P Emkey R D Effect of three years of oral alendronate treatment in postmenopausal women with osteoporosis. Am J Med 1996;101:488. 8948272 10.1016/s0002-9343(96)00282-3

[35] Black D M Cummings S R Karpf D B Randomised trial of effect of alendronate on risk of fracture in women with existing vertebral fractures. Fracture Intervention Trial Research Group. Lancet 1996;348:1535. 8950879 10.1016/s0140-6736(96)07088-2

[36] Cranney A Wells G Willan A Meta-analyses of therapies for postmenopausal osteoporosis. II. Meta-analysis of alendronate for the treatment of postmenopausal women. Endocr Rev 2002;23:508. 12202465 10.1210/er.2001-2002

[37] Bone H G Hosking D Devogelaer J P Ten years’ experience with alendronate for osteoporosis in postmenopausal women. N Engl J Med 2004;350:1189. 15028823 10.1056/NEJMoa030897

[38] Heaney R P Zizic T M Fogelman I Risedronate reduces the risk of first vertebral fracture in osteoporotic women. Osteoporos Int 2002;13:501. 12107665 10.1007/s001980200061

[39] Harris S T Watts N B Genant H K Effects of risedronate treatment on vertebral and nonvertebral fractures in women with postmenopausal osteoporosis: a randomized controlled trial. Vertebral Efficacy With Risedronate Therapy (VERT) Study Group. JAMA 1999;282:1344. 10527181 10.1001/jama.282.14.1344

[40] Sorensen O H Crawford G M Mulder H Long-term efficacy of risedronate: a 5-year placebo-controlled clinical experience. Bone 2003;32:120. 12633783 10.1016/s8756-3282(02)00946-8

[41] Chesnut C H III Skag A Christiansen C Effects of oral ibandronate administered daily or intermittently on fracture risk in post-menopausal osteoporosis. J Bone Miner Res 2004;19:1241. 15231010 10.1359/JBMR.040325

[42] Pyon E Y Once-monthly ibandronate for postmenopausal osteoporosis: review of a new dosing regimen. Clin Ther 2006;28:475. 16750461 10.1016/j.clinthera.2006.04.006

[43] US Food and Drug Administration Highlights of prescribing information - Boniva. www.accessdata.fda.gov/drugsatfda_docs/label/2011/021455s011lbl.pdf (Accessed on March 29, 2012).

[44] Reginster J Y Adami S Lakatos P Efficacy and tolerability of once-monthly oral ibandronate in postmenopausal osteoporosis: 2 year results from the MOBILE study. Ann Rheum Dis 2006;65:654. 16339289 10.1136/ard.2005.044958 PMC1798147

[45] Lyles K W Colón-Emeric C S Magaziner J S Zoledronic acid and clinical fractures and mortality after hip fracture. N Engl J Med 2007;357:1799. 17878149 10.1056/NEJMoa074941 PMC2324066

[46] Hadji P Papaioannou N Gielen E Persistence, adherence, and medication-taking behavior in women with postmenopausal osteoporosis receiving denosumab in routine practice in Germany, Austria, Greece, and Belgium: 12-month results from a European non-interventional study. Osteoporos Int 2015 10;26:2479–89. 26018090 10.1007/s00198-015-3164-4 PMC4575374

[47] Leder B Z Tsai J N Neer R M Response to Therapy With Teriparatide, Denosumab, or Both in Postmenopausal Women in the DATA (Denosumab and Teriparatide Administration) Study Randomized Controlled Trial. J Clin Densitom. 2016 Jul-Sep;19:346–51. 26900146 10.1016/j.jocd.2016.01.004

[48] Leder B Z Tsai J N Uihlein A V Denosumab and teriparatide transitions in postmenopausal osteoporosis (the DATA-Switch study): extension of a randomised controlled trial. Lancet 2015;386:1147–55. 26144908 10.1016/S0140-6736(15)61120-5 PMC4620731

[49] Cauley J A Norton L Lippman M E Continued breast cancer risk reduction in postmenopausal women treated with raloxifene: 4-year results from the MORE trial. Multiple outcomes of raloxifene evaluation. Breast Cancer Res Treat 2001;65:125–134. 11261828 10.1023/a:1006478317173

[50] Martino S Cauley J A Barrett-Connor E CORE Investigators Continuing outcomes relevant to Evista: breast cancer incidence in postmenopausal osteoporotic women in a randomized trial of raloxifene. J Natl Cancer Inst 2004;96:1751–61. 15572757 10.1093/jnci/djh319

[51] Chen J F Yang K H Zhang Z L A systematic review on the use of daily subcutaneous administration of teriparatide for treatment of patients with osteoporosis at high risk for fracture in Asia. Osteoporos Int 2015 1;26:11–28. 10.1007/s00198-014-2838-7. 25138261

[52] Harvey N C Kanis J A Odén A Efficacy of weekly teriparatide does not vary by baseline fracture probability calculated using FRAX. Osteoporos Int. 2015 9;26:2347–53. 26092062 10.1007/s00198-015-3129-7 PMC4532707

[53] Binkley N Bilezikian J P Kendler D L Official positions of the International Society for Clinical Densitometry and Executive Summary of the 2005 Position Development Conference. J Clin Densitom 2006;9:4. 16731426 10.1016/j.jocd.2006.05.002

[54] Grossman J M Gordon R Ranganath V K American College of Rheumatology 2010 recommendations for the prevention and treatment of glucocorticoid-induced osteoporosis. Arthritis Care Res (Hoboken) 2010;62:1515. 20662044 10.1002/acr.20295

[55] Saag K G Shane E Boonen S Teriparatide or alendronate in glucocorticoid-induced osteoporosis. N Engl J Med 2007;357:2028. 18003959 10.1056/NEJMoa071408

[56] Langdahl B L Marin F Shane E Teriparatide versus alendronate for treating glucocorticoid-induced osteoporosis: an analysis by gender and menopausal status. Osteoporos Int 2009;20:2095. 19350340 10.1007/s00198-009-0917-y

[57] Fuleihan G-H Salamoun M Mourad Y A Pamidronate in the prevention of chemotherapy-induced bone loss in premenopausal women with breast cancer: a randomized controlled trial. J Clin Endocrinol Metab 2005;90:3209. 15769994 10.1210/jc.2004-1444

[58] Greenspan S L Bhattacharya R K Sereika S M Prevention of bone loss in survivors of breast cancer: A randomized, double-blind, placebo-controlled clinical trial. J Clin Endocrinol Metab 2007;92:131. 17047022 10.1210/jc.2006-1272

[59] Vehmanen L Saarto T Risteli J Short-term intermittent intravenous clodronate in the prevention of bone loss related to chemotherapy-induced ovarian failure. Breast Cancer Res Treat 2004;87:181. 15377842 10.1023/B:BREA.0000041624.00665.4e

[60] Watts N B Adler R A Bilezikian J P Osteoporosis in men: an Endocrine Society clinical practice guideline. J Clin Endocrinol Metab 2012;97:1802. 22675062 10.1210/jc.2011-3045

[61] Zelissen P M Croughs R J van Rijk P P Raymakers J A Effect of glucocorticoid replacement therapy on bone mineral density in patients with Addison disease. Ann Intern Med 1994;120:207. 8273983 10.7326/0003-4819-120-3-199402010-00005

[62] www.rcplondon.ac.uk/pubs/books/glucocorticoid (Accessed on January 17, 2011).

